# Non-clonal occurrence of *pmrB* mutations associated with polymyxin resistance in carbapenem-resistant *Klebsiella pneumoniae* in Brazil

**DOI:** 10.1590/0074-02760180555

**Published:** 2019-05-20

**Authors:** Ana Claudia Souza Rodrigues, Ivson Cassiano de Oliveira Santos, Caroline Conci Campos, Isadora Nascimento Rezende, Yanara Miranda Ferreira, Claudia Elisabeth Volpe Chaves, Cláudio Marcos Rocha-de-Souza, Ana Paula D’Alincourt Carvalho-Assef, Marilene Rodrigues Chang

**Affiliations:** 1 Universidade Federal de Mato Grosso do Sul, Faculdade de Medicina, Programa de Pós-Graduação em Saúde e Desenvolvimento na Região Centro-Oeste, Campo Grande, MS, Brasil; 2 Universidade Anhanguera Uniderp, Faculdade de Medicina, Campo Grande, MS, Brasil; 3 Fundação Oswaldo Cruz-Fiocruz, Instituto Oswaldo Cruz, Laboratório de Pesquisa em Infecção Hospitalar, Rio de Janeiro, RJ, Brasil; 4 Universidade Federal de Mato Grosso do Sul, Faculdade de Ciências Farmacêuticas, Alimentos e Nutrição, Campo Grande, MS, Brasil; 5 Hospital Regional de Mato Grosso do Sul, Campo Grande, MS, Brasil

**Keywords:** colistin, polymyxins, multidrug resistance

## Abstract

**BACKGROUND:**

Polymyxins are currently used as a “last-line” treatment for multidrug-resistant Gram-negative infections.

**OBJECTIVES:**

To identify the major mechanisms of resistance to polymyxin and compare the genetic similarity between multi-drug resistant *Klebsiella pneumoniae* strains recovered from inpatients of public hospitals in the Mid-West of Brazil.

**METHODS:**

97 carbapenems non-susceptible *K. pneumoniae* were studied. β-lactamases (*bla*_OXA-48_, *bla*_KPC_, *bla*_NDM_, *bla*_CTX-M_, *bla*_SHV_, *bla*_TEM_, *bla*_IMP_, *bla*_VIM_) and *mcr*-1 to *mcr*-5 genes were investigated by polymerase chain reaction (PCR). Mutations in chromosomal genes (*pmrA*, *pmrB*, *phoP*, *phoQ*, *and mgrB*) were screened by PCR and DNA sequencing. Clonal relatedness was established by using pulsed-field gel electrophoresis and multilocus sequence typing.

**FINDINGS:**

*K. pneumoniae* isolates harbored *bla*_KPC_ (93.3%), *bla*_SHV_ (86.6%), *bla*_TEM_ (80.0%), *bla*_CTX-M_ (60%) genes. Of 15 *K. pneumoniae* resistant to polymyxin B the authors identified deleterious mutations in *pmrB* gene, mainly in T157P. None *K. pneumoniae* presented *mcr* gene variants. Genetic polymorphism analyses revealed 12 different pulsotypes.

**MAIN CONCLUSIONS:**

Deleterious mutations in *pmrB* gene is the main chromosomal target for induction of polymyxin resistance in carbapenem-resistant *K. pneumoniae* in public hospitals in the Mid-West of Brazil.

The emergence of carbapenem-resistant *Klebsiella pneumoniae* (CRKp) and the increased use of polymyxin B to treat infections caused by these microorganisms may have contributed to the spread of polymyxin-resistant *K*. *pneumoniae* isolates (PRKP).^1^ ^,^ ^2^ ^,^ ^3^ The polymyxin resistance is most commonly associated with the modification of the lipopolysaccharide (LPS) following the addition of 4-amino-4-deoxi-L-arabinose to lipid A. Modifications of Ara4N are regulated by two component systems: PhoP/PhoQ, PmrA/PmrB and MgrB regulator. Mutations in genes involved in the production of these systems may result in lower antibiotic fixation.^4^ Previous studies reported that disruption of *mgrB* gene is one of the major mechanisms of polymyxin resistance in *K. pneumoniae*.^2^ ^,^ ^5^ ^,^ ^6^

Recently, *mcr-1* gene and its variants (*mcr*-1 to *mcr*-8 genes) was described conferring resistance to polymyxin in *Enterobacteriaceae* isolated from humans, animals and environmental samples worldwide, including Brazil.^7^ ^,^ ^8^ ^,^ ^9^ The global dissemination of these genes may be a signal of a new era of pandrug resistant bacteria.^10^

To date, there is no information about the mechanisms of polymyxin resistance in Gram negative bacilli in Mato Grosso do Sul state. The aims of this study were to investigate the mechanisms of resistance to polymyxin and to evaluate the genetic diversity in carbapenem and polymyxin-resistant *K. pneumoniae* strains recovered from patients admitted in intensive care units of public hospitals in the Mid-West of Brazil.

## MATERIALS AND METHODS

*Bacterial isolates and susceptibility tests* - A total of 97 carbapenem non-susceptible *K. pneumoniae* isolated from patients admitted in intensive care units of three tertiary hospitals in Mato Grosso do Sul, Brazil (Hospital A, 592 beds; Hospital B, 271 beds and Hospital C, 352 beds) between 2013 and 2014 were included in this study. The identification and antimicrobial susceptibility testing were performed by Vitek 2 compact System (bioMérieux, Marcy L’Etoile, France). The minimum inhibitory concentration (MIC) of tigecycline was determined by using E-test ® strips (bioMérieux, Marcy L’Etoile, France) applied in Mueller-Hinton agar (Oxoid, England) according to the manufacturer’s instructions.

The polymyxin B MICs were performed by broth microdilution test according the CLSI.^11^ *Escherichia coli* ATCC 25922 and *Pseudomonas aeruginosa* ATCC 27853 were used as quality control. BrCast^12^ breakpoints were used for interpretation of tigecycline (Susceptible ≤ 1 mg/L, Resistant > 2 mg/L) and polymyxin MIC results (Susceptible ≤ 2 mg/L, Resistant > 2 mg/L).^12^ Multidrug-resistance (MDR) was defined as non-susceptibility to at least one agent in three or more antimicrobial categories. Extensively drug-resistant (XDR) was defined as susceptible to only one or two categories in all.^13^

*Screening for genes of resistance* - Resistant genes *bla*_OXA-48_-_like_, *bla*_KPC_, *bla*_NDM_ were investigate by multiplex polymerase chain reaction (PCR) and *bla*_CTX-M_, *bla*_SHV_, *bla*_TEM_, *bla*_IMP_, *bla*_VIM_ by simple PCR using primers as previously described.^14^ ^,^ ^15^ ^,^ ^16^ ^,^ ^17^ The plasmid-mediated polymyxin resistance gene*, mcr*-1 to *mcr*-5, was determined by multiplex PCR using primers as previously described.^7^

Mutations in chromosomal genes (*pmrA*, *pmrB*, *phoP*, *phoQ*, *and mgrB*) were screened by PCR and DNA sequencing.^18^ ^,^ ^19^ DNA was extracted from fresh bacterial colonies using an AxyPrep Bacterial Genomic DNA Miniprep kit (Axygen Scientific, Union city, CA, USA). The amplification products were purified using DNA Illustra GFX 96 kit (GE Healthcare Life Sciences, UK Ltd., Buckinghamshire, UK) and sequenced using the 3730 DNA analyser (Applied Biosystems, CA, USA). Data were analysed using Geneious (6.1.8) software (Auckland, New Zealand) and BLASTN (NCBI) tool (www.ncbi.nlm.nih.gov/blast). The PROVEAN platform was used to predict alterations in biological functions of proteins using *K. pneumoniae* MGH 78578 (CP00647.1) as reference.

*Genotyping by pulsed-field gel electrophoresis (PFGE) and multilocus sequence typing (MLST)* - Clonal relatedness among PRKp isolates was established using *Xba*I - PFGE (Promega, Charbonnières-les-Bains, France). DNA fragments were separated with a CHEF DR III apparatus (Bio-Rad; Richmond, CA - USA) and analysed by BioNumerics fingerprinting software (Applied Maths, Sint-Martens-Latem, Belgium).^20^

MLST was performed to subtype PRKp by amplification and sequenced of seven housekeeping genes (*gapA*, *infB*, *mdh*, *pgi*, *phoE*, *rpoB*, and *tonB*).^21^ The allelic profiles and sequence types (ST) were screened as determined by the Institute Pasteur *Klebsiella* MLST database (http://bigsdb.web.pasteur.fr/klebsiella/klebsiella.html).

*Ethics* - This study was approved by the Plataforma Brasil Research Ethics Committee.

## RESULTS

Of the 97 carbapenem non-susceptible *K. pneumoniae* isolates studied, 15 (15.5%) were resistant to polymyxin B (MIC > 2 mg/L) and nine of them had MIC ≥ 8 mg/L. The PRKp isolates were recovered from culture of ten urines (66.7%), two blood (13.3%), two scar tissue (13.3%) and one tracheal aspirate (6.7%).

Antimicrobial susceptibility test showed that seven PRKp were XDR. The lower resistance antibiotics were amikacin (6.7%), tigecycline (26.7%) and fosfomycin (33.3%). All PRKp were resistant to carbapenems (including 13.3% with MIC ≤ 8 μg/mL) and cephalosporins. Screening resistance gene showed that PRKp isolates harbored *bla*_SHV_ (86.6%), *bla*_TEM_ (80.0%), and *bla*_CTX-M_ (60%). Thirteen *K. pneumoniae* isolates contained three or more resistance genes and fourteen (93.3%) PRKp isolates carried the *bla*_KPC_ gene. The *bla*_OXA48_, *bla*_NDM_, *bla*_VIM_. *bla*_IMP_ and *mcr* genes variants were not detected.

Ten PRKp isolates presented the same amino acid substitution from threonine to proline at position 157 (T157P) in PmrB protein and four of these contained another mutation (R256G) that is considered deleterious by PROVEAN software. Two PRKp isolates (A38 and S378) presented others non-neutral mutation (H58N, A66E, V67D, A66E, P272H, G318A) in PmrB. No mutations in the *pmr*A gene nor in the PhoP/PhoQ System were observed. *K. pneumoniae* isolates exhibited non-neutral mutation in *mgrB* gene: A38B isolate with L19K mutation and S7 isolate with eight non-neutral mutation (V1G, K3V, L4P, W6S, V7D, L9K, I10N, V11K), including two insertions sequences. No mutation was associated with stop codon.

Genetic polymorphism analyses of PRKp revealed 12 different pulsotypes (A to L) by PFGE method with similarity below 85%. MLST analysis showed 11 ST among these isolates. ST 11 (belonging to CC258) was present in four PRKp isolates of all hospitals studied and ST13 in three PRKp, from two hospitals (A and C). The antimicrobial susceptibility profiles, resistance genes determinants and clonal patterns are shown in Table.

## DISCUSSION

Infections caused by multidrug-resistant *K. pneumoniae* are currently a concern in public health. Polymyxins are often the last line of therapeutic options for the treatment. Consequently, a high mortality rate has been observed, especially in intensive therapy units.^22^

It is well documented in the literature that the levels of resistance to antibiotics may vary according to the hospital characteristics and distinct geographic areas. In our study, PRKp isolates showed low resistance to tigecycline, fosfomycin, and amikacin suggesting these antibiotics would be successful as treatments in infections caused by carbapenem and polymyxin-resistant *K. pneumoniae*.

The results of this study show different mechanisms of antimicrobial resistance, including non-neutral mutations in *pmrB* gene (A66E, P272H, G318A, H58N, V67D) not previously associated to polymyxin resistance in *K. pneumoniae*.

Similar to previous Brazilian studies,^1^ ^,^ ^2^ ^,^ ^23^ we also observed a high rate KPC-producing *K. pneumoniae* and described high genetic diversity among the isolates.

**TABLE:** Polymyxin B minimum inhibitory concentrations (MICs) and molecular profile of carbapenem-resistant *Klebsiella pneumoniae* (CRKp) isolates in Mid-West region of Brazil

Isolate	Clinical specimen	Hospital	Resistance	Polymyxin B MIC (µg/mL)	Modification in protein					β-lactamases	PFGE	ST
					MgrB	PmrA	PmrB	PhoP	PhoQ			
S7	Urine	A	MDR: AMP, ATM, CMP, CFO, CAZ, CRO, CFZ, ERT, IMP, MPM, PPT, TIG, SUT	32	V1G*, insertion G in 4 and 5, K2R/D , K3V(*), L4P(*), R5K(-) , W6S(*), V7D(*), L9K(*), insertion C in 29 and 30, I10N(*), V11K(*), I12P(-)	WT	WT	WT	WT	KPC, CTX-M, SHV, TEM	G	11
S87	Scar tissue	A	MDR: AMP, ATM, CMP, CFO, CAZ, CRO, CFZ, CIP, ERT, IMP, MPM, PPT, TIG	32	I12V(-), T40A(-)	WT	T157P (*), R256G (*)	WT	WT	KPC, CTX-M, SHV, TEM	C	1298
S275	Blood	A	XDR: AMP, ATM, CMP, CFO, CAZ, CRO, CFZ, CIP, FOSF, ERT, IMP, MPM, PPT, SUT	64	WT	WT	T157P (*)	R34K	WT	KPC, CTX-M, SHV, TEM	H	2687
S336	Scar tissue	A	MDR: AMP, ATM, CMP, CFO, CAZ, CRO, CFZ, ERT, IMP, MPM, PPT, SUT	4	WT	WT	T157P (*) M175V (-), T246 A	WT	WT	KPC, CTX-M, TEM	J	13
S371	Urine	A	MDR: AMP, CMP, CFO, CAZ, CRO, CFZ, CIP, PPT, ERT, IMP, MPM	16	WT	WT	T157P (*), A228T (-), Q232E (-), I242V (-), N244S (-), E272Q (-), Q356R (-), T8N (-), N105S (-)	R34K	R69K, O92K, A106T E112D I139V, L163F, V196I, T372S H410Y O424L O482L O487E	KPC, SHV, TEM	B	1075
S378	Urine	A	MDR: AMP, ATM, CMP, CFO, CAZ, CRO, CFZ, CIP, ERT, IMP, MPM, SUT	8	WT	WT	H58N (*), A66E (*), V67D(*),A2G(-),T8N(-), L80M (-), M86V (-), N105S (-)	WT	WT	KPC, CTX-M, SHV, TEM	A	449
S400	Urine	A	XDR: AMP, ATM, CMP, CFO, CAZ, CRO, CFZ, CIP, ERT, FOSF, IMP, MPM, PPT, SUT	16	WT	WT	T157P (*)	WT	WT	KPC, CTX-M, SHV, TEM	M	70
S419	Urine	A#	XDR: AMI, AMP, ATM, CMP, CFO, CAZ, CRO, CFZ, CIP, GEN, ERT, IMP, MPM, PPT, TIG, SUT	16	WT	WT	T157P (*), M175V (-), T246A (-)	WT	WT	CTX-M, SHV, TEM	J	13
R86	Urine	C	MDR: AMP, ATM, CMP, CFO, CAZ, CRO, CFZ, CIP, ERT, IMP, MPM, PPT, SUT	4	WT	WT	T157P (*)	WT	WT	KPC	L	13
R98	Urine	C	XDR: AMP, ATM, CMP, CFO, CAZ, CRO, CFZ, CIP, FOSF, GEN, ERT, IMP, MPM, PPT, SUT	4	WT	WT	T157P (*)	WT	WT	KPC, SHV	G	11
A38	Blood	B	XDR: AMP, ATM, CMP, CFO, CAZ, CRO, CFZ, CIP, FOSF, GEN, ERT, IPM, MPM, PPT, SUT	8	V1L/M, L19K*	WT	A66E (*), P272H (*), G318A(*),A21P(-),L28V (-), Q41E (-), A47V(-),H61L (-), T247A (-), A290S (-), E605D (-), V327I (-)	WT	WT	KPC, TEM, SHV	E	273
A40	Tracheal aspirate	B	MDR: AMP, ATM, CMP, CAZ, CRO, CFZ, CIP, IMP, MPM, PPT, SUT	16	WT	WT	T157P (*)	WT	WT	KPC, SHV, TEM	D	2084
A58	Urine	B	XDR: AMP, ATM, CMP, CFO, CAZ, CRO, CFZ, CIP, ERT, FOSF, IPM, MPM, PPT, SUT	4	WT	WT	T246A (-), R256G (*)	L12M	WT	KPC, CTX-M, SHV	I	11
A61	Urine	B	XDR: AMP, ATM, CMP, CTX, CFO, CAZ, CRO, CFZ, CIP, GEN, ERT, IMP, PPT, TIG, SUT	4	WT	WT	T157P (*), R256G (*)	WT	M34R	KPC, TEM, SHV	F	323
A70	Urine	B	MDR: AMP, ATM, CMP, CFO, CAZ, CRO, CFZ, CIP, ERT, IMP, MPM, PPT, SUT	4	WT	WT	T157P (*), R256G (*)	WT	WT	KPC, CTX-M, SHV, TEM	I	11

MDR: multidrug-resistant; XDR: extensively drug-resistant; WT: wild type; AMI: amikacin; AMP: ampicillin; ATM: aztreonam; CMP: cefepime; CFO: cefoxitin; CAZ: ceftazidime; CRO: ceftriaxone; CFZ: cefazolin; CIP: ciprofloxacin; ERT: ertapenem; FOSF: fosfomycin; GEN: gentamicina; IMP: imipenem; MPM: meropenem; PPT: piperacillin/tazobactam; TIG: tigecycline; SUT: trimethoprim/sulfamethoxazole; PFGE: pulsed-field gel electrophoresis; MLST: multilocus sequence type; ST: sequence type. (*): mutation predicted as deleterious by PROVEAN. (-): mutation predicted as neutral by PROVEAN.

Almost 50% of *K. pneumoniae* studied was considered *K. pneumoniae* XDR. The high resistance to β-lactams observed (Table) may be related to the presence of genes as *bla*_SHV_, *bla*_TEM_ and *bla*_CTX-M_, mainly *bla*_KPC_.

Recent Brazilian studies carried out in the Southeast region of Brazil demonstrate a temporal increase of resistance to polymyxin since 2009, ranging from 0 to 30.6%.^2^ ^,^ ^3^ ^,^ ^24^ In our study, we documented that 15.5% of *K. pneumoniae* isolated between 2013 and 2014 were resistant to polymyxin, in the Brazilian Mid-West hospitals. This is the first description of resistance to polymyxin in this region.

The genetic analysis demonstrates that in these hospitals the resistance to polymyxin are from chromosomal origin because none *K. pneumoniae* presented *mcr* gene variants.

Although previous studies report that alterations in the *mgrB* gene is the main mechanisms of polymyxin resistance in Brazil and worldwide,^2^ ^,^ ^5^ ^,^ ^6^ ^,^ ^25^ ^,^ ^26^ in our study, only two PRKp presented deleterious mutations in this gene. Aires et al.^6^ reported disruption in *mgrB* gene by IS903B, IS5, IS102, ISKpn26 (IS5 family), and IS10L (IS4 family) in different regions of Brazil, including two *K. pneumoniae* from the Mid-West region (Distrito Federal).

Different from what was expected the majority of PRKp isolates studied carried alterations in *pmrB* gene, mainly in T157P. To be best of our knowledge, this kind of alteration has not been described in Brazil prior to this study. Jayol et al.^19^ showed this point mutation in the *pmrB* gene (T157P) leads to an upregulation to pmrCAB and pmrHFIJKLM operons conferring resistance to polymyxins in *K. pneumoniae* isolates from South-Africa, Colombia and Turkey.^19^

In our study, we identified four PRKp with non-neutral mutation (R256G) on PmrB protein as also described by Aires et al.^6^ in the Southeast region of Brazil^6^ Pitt et al.^26^ detected R256G in polymyxin-susceptible and in polymyxin-resistant *K. pneumoniae*, suggesting that this mutation is not determinant for polymyxin resistance. The same authors related simultaneous alterations in *pmr*B (P158R, T140P) and in *mgr*B genes with increases the MIC values.

In the hospitals studied, no relationship was observed between MIC values and mutations in *pmrB* genes. However, in *K. pneumoniae* with mutations in *mgrB* gene we observed high level polymyxin-resistant (MIC = 32 µg/mL), as also described by Aires et al.^6^

The most prevalent mechanism of resistance to polymyxin observed in the Mid-West region is due to mutation in the *pmrB* gene, different from that observed (mutation in the *mgr*B gene) in other Brazilian states.^1^ ^,^ ^2^ ^,^ ^6^ Antimicrobial pressures may be responsible for the evolution of different mutation profiles observed between different geographic regions.

Our study reveals high clonal diversity among PRKp isolates including nine different ST’s (ST13, ST70, ST273, ST449, ST323, ST2084, ST1075, ST1298, ST2687) that were not associated to polymyxin resistance previously. ST11 (CC258) was the most prevalent sequence type, followed by ST13. ST11 clone has been detected worldwide as the main international high-risk clones of *K. pneumoniae* associated with outbreaks and dissemination of carbapenemases and polymyxin resistance.^1^ ^,^ ^2^ ^,^ ^4^ ^,^ ^6^ ^,^ ^27^ Braun et al.^2^ described ST258 and ST437 (both belonging to 258 clonal complex) as the main sequence types in PRKp isolated from São Paulo, Brazil.

The emergence and evolution of complex 258 in KPC-*K. pneumoniae* is carried by mobile transposable elements. Pereira et al.^1^ described the clonal diversity in Brazil with prevalence of ST11 and ST340 in Northeast region, ST437 in South and Southeast regions and ST11 in Mid-West.

Our results suggest that PRKp isolates belonging to ST11 and ST13 clones are adapted in Mid-West region and highlights the emergence of new ST in PRKp (ST70, ST273, ST449, ST323, ST2084, ST1075, ST1298, ST2687). Complementation assays should be done later to elucidate the role of these STs and polymyxin resistance.

*In conclusion* - Our results show that many *K. pneumoniae* not susceptible to carbapenem isolated in Brazilian Mid-West Hospitals are considered MDR and XDR, but still show low resistance to tigecycline, fosfomycin and amikacin. The high resistance to β-lactams observed may be related to the presence of genes as *bla*_SHV_, *bla*_TEM_ and *bla*_CTX-M_, mainly *bla*_KPC_.

The resistance to polymyxin are from chromosomal origin because none *K. pneumoniae* presented *mcr* gene variants. The main mechanism of resistance to polymyxin in *K. pneumoniae* in the hospitals studied is due to a mutation in the *pmrB* gene.
